# Kinetics of nitrous oxide (N_2_O) formation and reduction by *Paracoccus pantotrophus*

**DOI:** 10.1186/s13568-016-0258-0

**Published:** 2016-10-03

**Authors:** B. L. Read-Daily, F. Sabba, J. P. Pavissich, R. Nerenberg

**Affiliations:** 1 Department of Engineering and Physics, Elizabethtown College, Elizabethtown, PA 17022 USA; 2 Department of Civil Engineering and Environmental Engineering and Earth Sciences, University of Notre Dame, Notre Dame, IN 46556 USA; 3 Facultad de Ingeniería y Ciencias, Universidad Adolfo Ibáñez, Avenida Padre Hurtado 750, Viña del Mar, Chile

**Keywords:** *Paracoccus pantotrophus*, Nitrous oxide, Denitrification, Maximum specific reduction rates, Kinetics

## Abstract

Nitrous oxide (N_2_O) is a powerful greenhouse gas emitted from wastewater treatment, as well as natural systems, as a result of biological nitrification and denitrification. While denitrifying bacteria can be a significant source of N_2_O, they can also reduce N_2_O to N_2_. More information on the kinetics of N_2_O formation and reduction by denitrifying bacteria is needed to predict and quantify their impact on N_2_O emissions. In this study, kinetic parameters were determined for *Paracoccus pantotrophus*, a common denitrifying bacterium. Parameters included the maximum specific reduction rates, $\hat{q}$, growth rates, ${\hat{\upmu }}$, and yields, Y, for reduction of NO_3_^−^ (nitrate) to nitrite (NO_2_^−^), NO_2_^−^ to N_2_O, and N_2_O to N_2_, with acetate as the electron donor. The $\hat{q}$ values were 2.9 gN gCOD^−1^ d^−1^ for NO_3_^−^ to NO_2_^−^, 1.4 gN gCOD^−1^ d^−1^ for NO_2_^−^ to N_2_O, and 5.3 gN gCOD^−1^ d^−1^ for N_2_O to N_2_. The ${\hat{\upmu }}$ values were 2.7, 0.93, and 1.5 d^−1^, respectively. When N_2_O and NO_3_^−^ were added concurrently, the apparent (extant) kinetics, $\hat{q}_{\text{app}}$, assuming reduction to N_2_, were 6.3 gCOD gCOD^−1^ d^−1^, compared to 5.4 gCOD gCOD^−1^ d^−1^ for NO_3_^−^ as the sole added acceptor. The ${\hat{\upmu }}_{\text{app}}$ was 1.6 d^−1^, compared to 2.5 d^−1^ for NO_3_^−^ alone. These results suggest that NO_3_^−^ and N_2_O were reduced concurrently. Based on this research, denitrifying bacteria like *P. pantotrophus* may serve as a significant sink for N_2_O. With careful design and operation, treatment plants can use denitrifying bacteria to minimize N_2_O emissions.

**Electronic supplementary material:**

The online version of this article (doi:10.1186/s13568-016-0258-0) contains supplementary material, which is available to authorized users.

## Introduction

Nitrous oxide (N_2_O) is a potent greenhouse gas with a global warming potential 300-fold greater than CO_2_ (IPCC 2006). It also is a major concern for ozone depletion in the stratosphere (Ravishankara et al. 2009). In recent years, wastewater treatment processes, especially those employing biological nutrient removal (BNR), have been found to be significant sources of N_2_O (Ni and Yuan 2015). The most common sources of N_2_O in BNR processes are ammonium-oxidizing bacteria (AOB) and heterotrophic denitrifying bacteria (DNB) (Law et al. 2012). AOB can form significant amounts of N_2_O, especially when the dissolved oxygen (DO) concentrations are low, or during transitions from anoxic to aerobic conditions (Chandran et al. 2011; Sabba et al. 2015). During denitrification, N_2_O can form when insufficient electron donor is available, when the pH is excessively high, when sufficient copper is lacking, or when inhibitors of the N_2_O reductase, such as DO, hydrogen sulfide, high nitrite (${\text{NO}}_{2}^{ - }$) or ammonia (NH_3_) concentrations, are present (Tallec et al. 2008; Bergaust et al. 2010; Lu and Chandran 2010; Pan et al. 2012, 2013a).

While DNB can be a source of N_2_O emissions, they also can scavenge N_2_O and reduce it to N_2_ (Zumft and Kroneck 2007). For example, N_2_O produced by nitrifying bacteria can be reduced by DNB in the anoxic zone of a suspended-growth process or in the deeper portions of a biofilm (Ikeda-Ohtsubo et al. 2013).

A better understanding, and quantification, of the kinetics of N_2_O reduction by DNB is critical to predicting N_2_O emissions from wastewater treatment processes and developing strategies for N_2_O mitigation. Since N_2_O reduction may take place in the presence of ${\text{NO}}_{3}^{ - }$, it also is important to explore the kinetics when both acceptors are present (Schreiber et al. 2012). These parameters are needed for more recent mathematical models that explicitly include N_2_O as a state variable, such as those developed by (Ni and Yu 2008; Hiatt and Grady 2008; Ni et al. 2011; Pan et al. 2013b).

In this research, we determined denitrification kinetics of a pure culture of *Paracoccus pantotrophus* (formerly *Thiosphaera pantotropha*), a versatile denitrifying bacterium isolated from denitrifying wastewater treatment processes (Robertson and Kuenen 1983). We used a multistep model including the reduction of ${\text{NO}}_{3}^{ - }$ to ${\text{NO}}_{2}^{ - }$, ${\text{NO}}_{2}^{ - }$ to N_2_O, and N_2_O to N_2_, and determined the biomass yield (Y), $\hat{q}$, and maximum growth rate (${\hat{\upmu }}$) for each step. We also determined the apparent $\hat{q}$ and ${\hat{\upmu }}$, based solely on donor oxidation and biomass formation, for the reduction of ${\text{NO}}_{3}^{ - }$ to N_2_ and concurrent reduction of ${\text{NO}}_{3}^{ - }$ and N_2_O. Our objective was to gain a better understanding of the mechanisms of N_2_O formation and reduction by DNB.

## Materials and methods

### Bacterial strain and growth medium

We used a pure culture of *P. pantotrophus* (ATCC 35512) in this study. A minimal growth medium was used, consisting of 1.386 g Na_2_HPO_4_, 0.849 g KH_2_PO_4_, 0.02 g MgSO_4_·7H_2_O, and 0.1 g (NH_4_)_2_SO_4_, 0.1 mL Ca–Fe solution, and 0.1 mL trace mineral solution (Nerenberg et al. 2002). The medium also included a trace amount of Luria–Bertani (LB) broth, at 1 % of the usual concentration, to minimize microbial aggregation during growth. All chemicals were analytical grade. Nitrogen gas was UHP grade and ${\text{NO}}_{3}^{ - }$ was added as needed to obtain the desired initial concentrations. N_2_O gas was 99.5 % purity and was added into the headspace.

### Batch studies

Batch tests were carried out in 1-L glass bottles with 200 mL of minimal medium. Bottles were capped with a cored rubber stopper containing a sectioned Balch tube with a butyl rubber stopper and aluminum crimp seal, allowing for sample collection. Bottles were successively vacuum-degassed to −1.7 atm and pressurized with either N_2_ or N_2_O at 1.3 atm, three times. The final headspace contained either N_2_ or N_2_O at 1.3 atm. Batch tests were carried out at least in triplicate.

Bottles were inoculated with 100 µL of *P. pantotrophus* culture with an optical density at 600 nm (OD_600_) of 0.6. Bottles were shaken on their sides at 150 rpm at room temperature (22 °C). The medium was amended with acetate as an electron donor and carbon source, with an initial concentration of 650 mgCOD L^−1^ (600 mg/L as acetate). When ${\text{NO}}_{3}^{ - }$ was used, its initial concentration was 50 mgN L^−1^.

### Analytical methods

Acetate, ${\text{NO}}_{3}^{ - }$, and ${\text{NO}}_{2}^{ - }$ were analyzed using a Dionex ICS2500 ion chromatograph (IC, Dionex Corporation, Sunnyvale, CA) with a 4-mm Dionex AS-11 column, an AG-11 guard column, and a conductivity detector. The program consisted of a 5-min equilibration with 4 mM sodium hydroxide eluent, injection of the sample, a 9-min isocratic run at 4 mM, and a linear gradient from 4 to 50 mM sodium hydroxide over 2 min. A Dionex ASRS suppressor was used in internal recycle mode. Injection was performed with a Dionex AS40 automated sampler. The injection volume was 200 μL. The detection limit for acetate, ${\text{NO}}_{3}^{ - }$, and ${\text{NO}}_{2}^{ - }$ was approximately 0.1 mgN L^−1^. The biomass concentration was assessed with a spectrophotometer via the OD_600_ (UV10, Thermo, Rochester, NY) and converted to dry weight (DW) using a conversion factor. A conversion factor of 385 mgDW L^−1^ per OD unit was determined following (Nerenberg et al. 2006).

### Determination of parameters

The maximum specific growth rates, ${\hat{\upmu }}$ (d^−1^), maximum specific substrate utilization rates, $\hat{q}$ (gCOD gCOD^−1^ d^−1^ or gN gCOD^−1^ d^−1^), and yields, Y (gCOD gCOD^−1^ or gCOD gN^−1^), were determined by parameter fitting (Reichert et al. 1995; Wild et al. 1995). A three-step model was used, including (1) ${\text{NO}}_{3}^{ - }$ reduction to ${\text{NO}}_{2}^{ - }$, (2) ${\text{NO}}_{2}^{ - }$ reduction to N_2_O, and (3) N_2_O reduction to N_2_. The model lumped NO reduction together with ${\text{NO}}_{2}^{ - }$ reduction, as NO reduction to N_2_O is very fast and NO accumulation during denitrification is minimal (Schreiber et al. 2012).

The process matrix is shown in Table 1 while the model components and the kinetic and stoichiometric parameters are shown in Additional file 1: Tables S1 and S2. Since the ${\text{NO}}_{3}^{ - }$, N_2_O, and acetate concentrations were well above their expected half-saturation constants for essentially the entire duration of the tests, the half saturation constants K_s_ for ${\text{NO}}_{3}^{ - }$, ${\text{NO}}_{2}^{ - }$, N_2_O, and acetate were not determined experimentally. Values were taken from (Ni et al. 2011). The specific rate of decay coefficient, b, also was considered insignificant compared to the maximum growth rates and therefore not independently determined. The value for b was taken as 0.15 d^−1^ (Rittmann and McCarty 2001).

**Table 1:** Process matrix for denitrification model

Components reactions	S_NO3-N_ mgN L^−1^	S_NO2-N_ mgN L^−1^	S_N2O-N_ mgN L^−1^	S mgCOD L^−1^	X mgCOD L^−1^	Rate expression
Nitrate reduction (NAR, NAP)	$- \frac{{1 - Y_{{NO_{3}^{ - } }} }}{{1.14Y_{{NO_{3}^{ - } }} }}$	$\frac{{1 - Y_{{NO_{3}^{ - } }} }}{{1.14Y_{{NO_{3}^{ - } }} }}$		$\frac{ - 1}{{Y_{{NO_{3}^{ - } }} }}$	1	$\hat{q}_{{NO_{3}^{ - } }} \, \times \,Y_{{NO_{3}^{ - } }} \, \times \,\frac{{S_{{NO_{3}^{ - } }} }}{{K_{{NO_{3}^{ - } }} + S_{{NO_{3}^{ - } }} }}\, \times \,\frac{{S_{S} }}{{K_{S} + S_{S} }}\, \times \,X_{H}$
Nitrite reduction (NIR)		$- \frac{{1 - Y_{{NO_{2}^{ - } }} }}{{1.14Y_{{NO_{2}^{ - } }} }}$	$\frac{{1 - Y_{{NO_{2}^{ - } }} }}{{1.14Y_{{NO_{2}^{ - } }} }}$	$\frac{ - 1}{{Y_{{NO_{2}^{ - } }} }}$	1	$\hat{q}_{{NO_{2}^{ - } }} \, \times \,Y_{{NO_{2}^{ - } }} \, \times \,\frac{{S_{{NO_{2}^{ - } }} }}{{K_{{NO_{2}^{ - } }} + S_{{NO_{2}^{ - } }} }}\, \times \,\frac{{S_{S} }}{{K_{S} + S_{S} }}\, \times \,X_{H}$
Nitrous oxide reduction (N_2_OR)			$- \frac{{1 - Y_{{NO_{2}^{ - } }} }}{{0.57Y_{{NO_{2}^{ - } }} }}$	$\frac{ - 1}{{Y_{{NO_{2}^{ - } }} }}$	1	$\hat{q}_{{NO_{2}^{ - } }} \, \times \,Y_{{NO_{2}^{ - } }} \, \times \,\frac{{S_{{NO_{2}^{ - } }} }}{{K_{{NO_{2}^{ - } }} + S_{{NO_{2}^{ - } }} }}\, \times \,\frac{{S_{S} }}{{K_{S} + S_{S} }}\, \times \,X_{H}$
Cell decay					−1	$- b_{H} \, \times \,X_{H}$

The experimental strategy consisted of (1) determining the $\hat{q}$, Y, and $\hat{\mu }$ for N_2_O using batch tests with N_2_O as the sole added acceptor; (2) after incorporating the parameters for N_2_O into the denitrification model (Table 1), determining the $\hat{q}$, Y, and $\hat{\mu }$ for reduction of ${\text{NO}}_{3}^{ - }$ to ${\text{NO}}_{2}^{ - }$, as well as the $\hat{q}$ for reduction of ${\text{NO}}_{2}^{ - }$ to N_2_O, from batch tests with ${\text{NO}}_{3}^{ - }$ as the sole added acceptor. When ${\text{NO}}_{3}^{ - }$ was added, accumulation of ${\text{NO}}_{2}^{ - }$ occurred at values greatly exceeded the reported K_s_ for ${\text{NO}}_{2}^{ - }$, which typically are below 1 mgN L^−1^. This accumulation allowed the $\hat{q}$ value for ${\text{NO}}_{2}^{ - }$ reduction to be determined from the ${\text{NO}}_{3}^{ - }$ reduction test. The Y for reduction of ${\text{NO}}_{2}^{ - }$ to N_2_O, in gCOD/gCOD, was assumed to be the same as the Y for reduction of N_2_O to N_2_ (Hiatt and Grady 2008; Ni et al. 2011).

Tests were also carried out with ${\text{NO}}_{3}^{ - }$ plus N_2_O as concurrently added acceptors. For these tests, as well as for the previous tests with ${\text{NO}}_{3}^{ - }$ as the sole added acceptor, we determined apparent (extant) parameters $\hat{q}_{app}$, $Y_{app}$ and $\hat{\mu }_{app}$. These were determined solely from acetate oxidation and biomass growth data, without considering acceptor utilization. Thus, these parameters reflect the concurrent use of multiple acceptors. The model was adapted from Ni et al. (2011) implemented using AQUASIM (Reichert et al. 1995; Wild et al. 1995). Parameters were determined using AQUASIM’s parameter estimation function. Each batch test was carried out at least in triplicate. The reported values are the average and standard deviation.

## Results

### Parameters for partial reduction steps

**Fig. 1:**
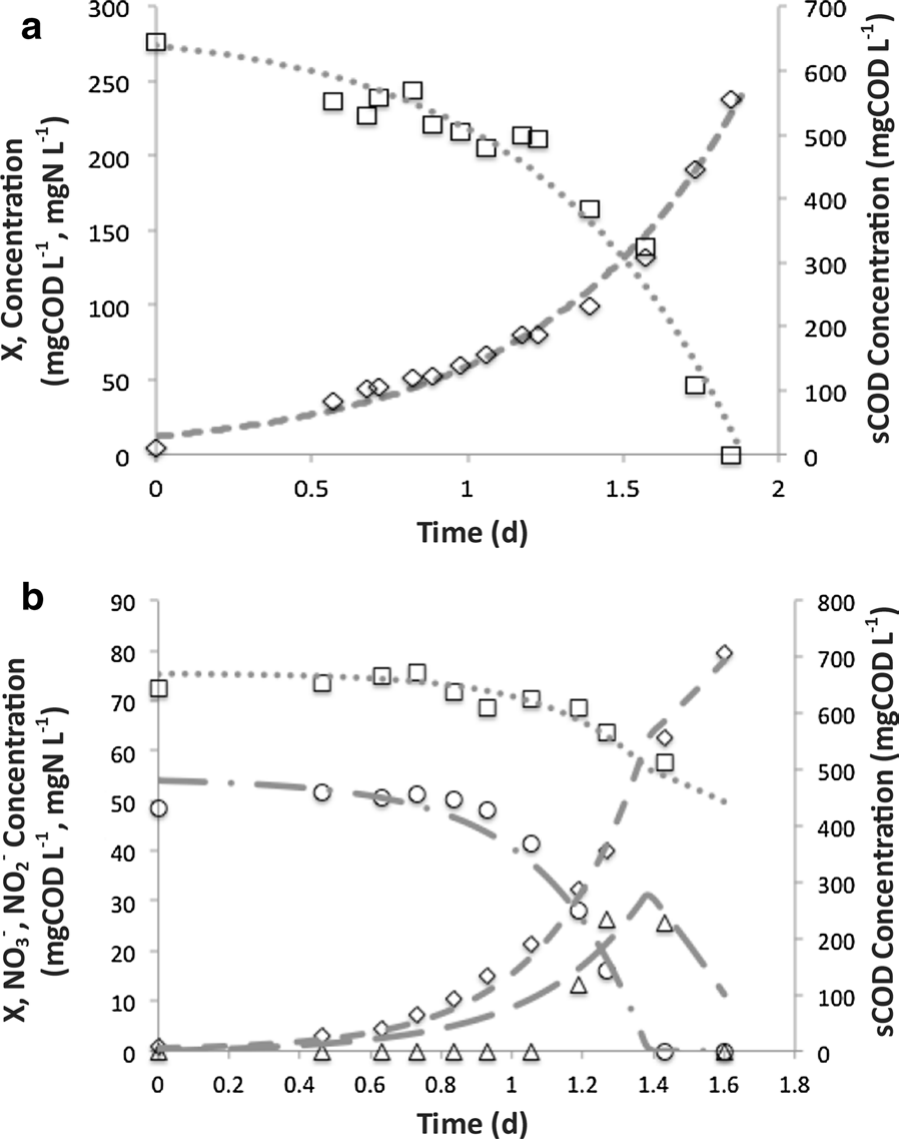
Typical batch and modeling (data fitting) results for **a** N_2_O as sole electron acceptor, **b** ${\text{NO}}_{3}^{ - }$ as sole added electron acceptor; model sCOD (*dotted line*), model biomass (), model ${\text{NO}}_{3}^{ - }$ (), model ${\text{NO}}_{2}^{ - }$ (), experimental sCOD (*square*), experimental biomass (*diamond*), experimental ${\text{NO}}_{3}^{ - }$ (*circle*), experimental ${\text{NO}}_{2}^{ - }$ (*triangle*)

Typical plots for the batch tests are shown in Fig. 1. The tests with N_2_O as the sole electron acceptor showed vigorous growth. Since one atmosphere of pure N_2_O gas was supplied in the headspace, and the bottles were vigorously shaken, the theoretical value of N_2_O in the aqueous phase was 905 mg L^−1^ and therefore non-rate-limiting. This was confirmed by the exponential growth observed throughout the tests with N_2_O as the sole acceptor. Because N_2_O was in excess, acetate was fully consumed during the experiment. In contrast, the tests with ${\text{NO}}_{3}^{ - }$ as the sole added electron acceptor had an initial ${\text{NO}}_{3}^{ - }$ concentration of only 50 mgN L^−1^. In these tests, acetate was only partially consumed and the final biomass concentration was much lower.

Data fitting was used to determine kinetic parameters from the experimental data. Parameters included the $\hat{\mu }$, $\hat{q}$, and Y for reduction of ${\text{NO}}_{3}^{ - }$ to ${\text{NO}}_{2}^{ - }$, ${\text{NO}}_{2}^{ - }$ to N_2_O, and N_2_O to N_2_. Results are summarized in Table 2. The $\hat{\mu }$ for ${\text{NO}}_{3}^{ - }$ reduction to ${\text{NO}}_{3}^{ - }$ was highest (2.7 d^−1^), and that for NO_2_^−^ reduction to N_2_O was the lowest (0.93 d^−1^). The $\hat{\mu }$ for N_2_O reduction (1.7 d^−1^) was lower than for ${\text{NO}}_{3}^{ - }$, but around double that for ${\text{NO}}_{3}^{ - }$. Note that these rates are for individual denitrification steps. The observed growth rates on ${\text{NO}}_{3}^{ - }$ or ${\text{NO}}_{3}^{ - }$, where the reduction products are utilized concurrently, would probably be higher.

**Table 2:** Summary of kinetic and stoichiometric parameters

Reactions	${\hat{\upmu }}$		$\hat{q}$		Y
	d^−1^	gCOD gCOD^−1^ d^−1^	gN gCOD^−1^ d^−1^	gCOD gCOD^−1^d^−1^	gCOD gN^−1^
${\text{NO}}_{3}^{ - }$ → ${\text{NO}}_{2}^{ - }$	2.7	6.0 ± 1.5	2.9 ± 0.72	0.45 ± 1.5	0.93 ± 0.72
${\text{NO}}_{2}^{ - }$ → N_2_O	0.93	2.6 ± 0.44	1.4 ± 0.25	0.36^a^	0.65
N_2_O → N_2_	1.7	4.8 ± 0.48	5.3 ± 0.27	0.36 ± 0.02	0.32 ± 0.27

^a^ ${\text{NO}}_{2}^{ - }$ yields were assumed to be the same as N_2_O

The $\hat{q}$ can be expressed in terms of the acceptor (gN gCOD d^−1^) or in terms of the donor (gCOD gCOD^−1^ d^−1^). The first is useful for identifying kinetic bottlenecks during sequential reduction of nitrogen oxides, as the downstream rate must be equal or higher than the upstream to avoid significant intermediate accumulation. The second is useful when assessing donor demand resulting from different combinations of acceptors. The two forms are related by stoichiometry.

In terms of N, the $\hat{q}$ for reduction of ${\text{NO}}_{3}^{ - }$ to ${\text{NO}}_{3}^{ - }$ was 2.9 gN gCOD d^−1^, and for reduction of ${\text{NO}}_{3}^{ - }$ to N_2_O was 1.4 gN g CODd^−1^ (Table 2). The $\hat{q}$ for reduction of N_2_O was highest at 5.3 gN gCOD d^−1^. When examining the COD oxidation results, the highest $\hat{q}$ was obtained for ${\text{NO}}_{3}^{ - }$ reduction to ${\text{NO}}_{3}^{ - }$, at 6.0 gCOD gCOD^−1^ d^−1^, consistent with its high growth rate. The $\hat{q}$ for ${\text{NO}}_{3}^{ - }$ reduction to N_2_O was only 2.6 gCOD gCOD^−1^ d^−1^, while N_2_O was 4.8 gCOD gCOD^−1^ d^−1^.

### Batch tests with concurrent addition of ${\text{NO}}_{3}^{ - }$ and N_2_O

**Fig. 2:**
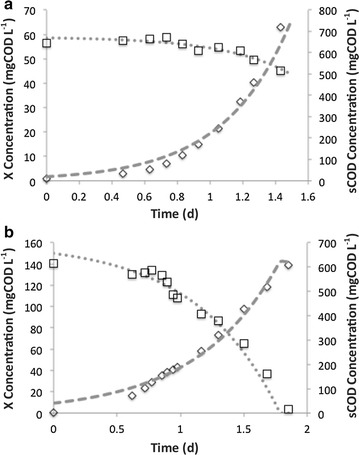
Typical batch tests for the determination of apparent rates for **a** ${\text{NO}}_{3}^{ - }$ and **b** ${\text{NO}}_{3}^{ - }$ plus N_2_O. Model sCOD (*dotted line*), model biomass (*dashed line*), experimental sCOD (*square*), experimental biomass (*diamond*)

Batch tests were used to compare the reduction rates of ${\text{NO}}_{3}^{ - }$, as the sole added acceptor, with rates of concurrently added ${\text{NO}}_{3}^{ - }$ and N_2_O. In order to explore the aggregate specific rates of growth and donor oxidation, the batch tests were fitted to determine the “apparent” or extant specific growth rates and donor utilization rates. Figure 2 shows the resulting plots and Table 3 summarizes the parameters. The combined addition of N_2_O and ${\text{NO}}_{3}^{ - }$ slowed the apparent $\hat{\mu }$ from 2.5 to 1.6 d^−1^. However, the apparent $\hat{q}$ increased from 5.4 to 6.3 gCOD gCOD^−1^ d^−1^.

**Table 3:** Summary of apparent parameters

Reactions	${\hat{\upmu }}_{app}$		$\hat{q}_{app}$		Y_app_
	d^−1^	gCOD gCOD^−1^ d^−1^	gN gCOD^−1^ d^−1^	gCOD gCOD^−1^ d^−1^	gN gCOD^−1^ d^−1^
${\text{NO}}_{3}^{ - }$ → N_2_	2.5 ± 0.96	5.4 ± 0.48	0.99 ± 0.09^a^	0.48 ± 0.09	2.6 ± 0.09^a^
${\text{NO}}_{3}^{ - }$ + N_2_O → N_2_	1.6 ± 0.11	6.3 ± 1.3	1.7 ± 0.34^a^	0.25 ± 0.03	0.95 ± 0.03^a^

^a^Calculated from donor utilization data, considering ${\text{NO}}_{3}^{ - }$ reduction to N_2_

## Discussion

Kinetic parameters for the denitrification pathway for *P. pantotrophus* were determined. The growth rates on N_2_O are high, suggesting that DNB can thrive when N_2_O is the sole electron acceptor. When ${\text{NO}}_{3}^{ - }$ and N_2_O are supplied together, the growth rates are higher than with N_2_O alone, but lower than with ${\text{NO}}_{3}^{ - }$ alone.

The lower $\hat{q}$ value for ${\text{NO}}_{2}^{ - }$ indicates a bottleneck on the denitrification pathway, i.e., when ${\text{NO}}_{3}^{ - }$ is present at non-rate-limiting concentrations, ${\text{NO}}_{2}^{ - }$ necessarily accumulates, and the observed rate of N_2_O reduction is limited to the maximum rate of N_2_O formation from ${\text{NO}}_{2}^{ - }$. Since the $\hat{q}$ for N_2_O, expressed as N, is around triple that of ${\text{NO}}_{2}^{ - }$ and almost double that of ${\text{NO}}_{3}^{ - }$, there appears to be significant capacity for N_2_O reduction concurrently with ${\text{NO}}_{3}^{ - }$ or ${\text{NO}}_{2}^{ - }$. In fact, our research shows that *P. pantotrophus* can concurrently utilize ${\text{NO}}_{3}^{ - }$ and N_2_O. Thus, DNB should be able to reduce externally supplied N_2_O concurrently with ${\text{NO}}_{3}^{ - }$ or ${\text{NO}}_{2}^{ - }$.

Few sets of kinetic data for the individual reduction steps have been previously reported. While some values have been reported for mixed culture (Additional file 1: Tables S3–S5), very few studies have assessed pure culture kinetics values. While environmental systems typically are based on mixed cultures, such mixed cultures are not reproducible and may give false indications of the mechanisms and regulation of denitrification. For example, for a given inoculum, a reduction test for N_2_O typically will be different from the community for a ${\text{NO}}_{3}^{ - }$ reduction test (Shade et al. 2013). The latter could select for bacteria that reduce ${\text{NO}}_{3}^{ - }$ to ${\text{NO}}_{2}^{ - }$ over denitrifiers, so ${\text{NO}}_{2}^{ - }$ accumulation would be due to microbial selection, not the intrinsic kinetics of a denitrifying system.

Values for $\hat{q}$ were reported by several researchers (von Schulthess et al. 1994; Wild et al. 1994; von Schulthess et al. 1995; Wild et al. 1995; Wicht 1996) (Additional file 1: Tables S3–S5). However, these values vary widely from 0.88 to 11.1 gN gCOD d^−1^ for a mixed culture grown on N_2_O (Additional file 1: Table S5). In other studies, $\hat{\mu }$ values were reported for growth on pure cultures of denitrifying bacteria using N_2_O as an acceptor, but not for ${\text{NO}}_{3}^{ - }$ to ${\text{NO}}_{2}^{ - }$ or ${\text{NO}}_{2}^{ - }$ to N_2_O (Strohm et al. 2007). The $\hat{\mu }$ for N_2_O in this study was 1.7 d^−1^, falling in the range that was previously reported for *P. denitrificans* (Koike and Hattori 1975), 1.37–2.57 d^−1^. The $\hat{q}$ values fall within the range of values previously reported for mixed cultures of denitrifying bacteria when N_2_O is reduced to N_2_. The yields on N_2_O presented in this paper are consistent with previous studies on the closely related DNB species *P. denitrificans* and *Pseudomonas stutzeri,* using acetate as an electron donor.

When examining the batch tests where N_2_O an ${\text{NO}}_{3}^{ - }$ were both supplied as electron acceptors, the results suggest that N_2_O was being reduced concurrently with ${\text{NO}}_{3}^{ - }$, leading to higher specific rates of donor utilization. The addition of N_2_O may have diverted electron equivalents from ${\text{NO}}_{3}^{ - }$ to N_2_O, which has a lower specific growth rate. This could lead to the lower overall apparent specific growth rate. Competition for electron carriers in DNB has been proposed by some researchers, who incorporated it in a metabolic model (Pan et al. 2013b, 2015). This approach has much greater complexity than conventional models, but may be warranted in cases where the donor oxidation rate is limiting (Pocquet et al. 2016).

The results from this study provide important insights into the mechanisms of N_2_O formation and consumption by denitrifying microorganisms. In particular, the parameters may be important for assessing the role of DNB in scavenging N_2_O produced by nitrifiers or due to incomplete denitrification (Sabba et al. 2015). N_2_O may be produced at a given time or location within a process, but could potentially be consumed at a different time or location by N_2_O-reducing microorganisms such as *P. pantotrophus*.

**Fig. 3:**
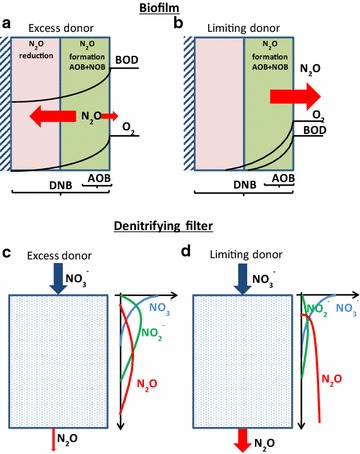
*Top panels* theoretical behavior of denitrifying bacteria in biofilms under (**a**) excess or (**b**) limiting electron donor conditions. *Lower panels* theoretical nitrogen profiles in a denitrifying filter in presence of (**c**) excess or (**d**) limiting electron donor

The role of DNB in producing and consuming N_2_O is illustrated schematically in Fig. 3. In Fig. 3a, a biofilm is supplied with ammonium, DO, and COD. N_2_O is formed by AOB, especially as the DO decreases, and some also is produced by the DNB. However, DNB provide a sink for N_2_O in the anoxic zone, so only a fraction of the produced N_2_O escapes to the bulk liquid (Sabba et al., submitted). If COD does not reach the base of the biofilm, little or no N_2_O will be reduced. Thus, all formed N_2_O will be released to the bulk (Fig. 3b). Another example is a denitrifying filter (Fig. 3c). If an influent containing COD and ${\text{NO}}_{3}^{ - }$ enters the top, ${\text{NO}}_{3}^{ - }$ is reduced first, with some ${\text{NO}}_{2}^{ - }$ and N_2_O accumulation. Then ${\text{NO}}_{2}^{ - }$ is reduced, and finally N_2_O is fully reduced towards the bottom. Again, if COD is limiting (Fig. 3d), N_2_O can break through the filter and be emitted to the environment. This breakthrough of N_2_O was recently demonstrated in a full-scale denitrifying filter (Bollon et al. 2016).

Our research suggests that, while DNB be a source of N_2_O, proper management of treatment conditions can allow DNB to scavenge N_2_O previously produced by AOB or DNB. This is especially true for biofilm systems or denitrifying filters, where zones of N_2_O formation may be adjacent to, or precede, zones where DNB can scavenge N_2_O. Providing anoxic conditions and sufficient electron donor is a key for effective N_2_O scavenging.

## Additional file

10.1186/s13568-016-0258-0 Additional tables.

## Abbreviations

AOB: ammonium oxidizing bacteria
BNR: biological nitrogen removal
CO_2_: carbon dioxide
COD: chemical oxygen demand
DNB: denitrifying bacteria
DW: dry weight
DO: dissolved oxygen
IC: ion chromatography
H_2_O: water
LB: Luria–Bertani
N_2_O: nitrous oxide
NH_3_: ammonia
NO: nitric oxide
$${\text{NO}}_{2}^{ - }$$: nitrite
OD: optical density
O_2_: oxygen
