# Supplementary material for: Kinetics of nitrous oxide (N2O) formation and reduction by Paracoccus pantotrophus
Source: AMB Express. 2016 Oct 3;6:85. doi: 10.1186/s13568-016-0258-0 (PMC5047877; doi:10.1186/s13568-016-0258-0)
Supplement: Supplementary file 1 — 10.1186/s13568-016-0258-0 Additional tables. [file 13568_2016_258_MOESM1_ESM.docx]

**Supplementary Information**

**Kinetics of Nitrous Oxide (N_2_O) Formation and Reduction during Denitrification**

*B.L. Read-Daily^1^, F. Sabba^2^, J.P. Pavissich^3^, R. Nerenberg^2^**

^1^Department of Engineering and Physics, Elizabethtown College, Elizabethtown, PA, 17022 USA

^2^College of Engineering and Science, Universidad Adolfo Ibáñez , Avenida Padre Hurtado 750 Viña del Mar, Chile

^3^Department of Civil and Environmental Engineering and Earth Science, University of Notre Dame, 156 Fitzpatrick Hall, Notre Dame, IN 46556 USA

***Corresponding author:**

Robert Nerenberg, Phone: +1 574 631 4098; fax +1 574 631 9236; e-mail: [rnerenbe@nd.edu](mailto:rnerenbe@nd.edu)

**The following are included as supporting information for this paper:**

Number of pages: 9

Number of tables: 5

**Table S1.** Model components

| **Variable** | **Description** | **Unit** |
| --- | --- | --- |
| **S_S_** | Acetate concentration | mgCOD L^-1^ |
| **S_NO3-_** | Nitrate concentration | mgN L^-1^ |
| **S_NO2-_** | Nitrite concentration | mgN L^-1^ |
| **S_N2O_** | Nitrous oxide concentration | mgN L^-1^ |
| **X_H_** | Active heterotrophic biomass concentration | mgCOD L^-1^ |

**Table S2.** Kinetic and stoichiometric parameters of the model

| **Variable** | **Description** | **Unit** |
| --- | --- | --- |
| $\hat{\boldsymbol{q}}$**_NO3-_** | Maximum nitrate reduction rate | gCOD gCOD^-1^d^-1^ |
| $\hat{\boldsymbol{q}}$**_NO2-_** | Maximum nitrite reduction rate | gCOD gCOD^-1^ d^-1^ |
| $\hat{\boldsymbol{q}}$**_N2O_** | Maximum nitrous oxide reduction rate | gCOD gCOD^-1^ d^-1^ |
| **K_NO3-_** | S_NO3_ affinity constant for Nar | mgN L^-1^ |
| **K_NO2-_** | S_NO2_ affinity constant for Nir | mgN L^-1^ |
| **K_N2O_** | S_N2O_ affinity constant for Nos | mgN L^-1^ |
| **Y_NO3-_** | Yield for heterotroph growth on NO_3_^-^ | gCOD gCOD^-1^ |
| **Y_NO2-_** | Yield for heterotroph growth on NO_2_^-^ | gCOD gCOD^-1^ |
| **Y_N2O_** | Yield for heterotroph growth on N_2_O | gCOD gCOD^-1^ |
| **b_H_** | Decay coefficient | d^-1^ |

**Table S3.** Kinetic parameters for NO_3_^-^ to NO_2_^-^ reduction by mixed and pure cultures of denitrifying bacteria

| **Type of Bacteria** | $\hat{\boldsymbol{\mu}}$**_NO3-_**  d^-1^ | **Y_NO3-_**  gCOD gN^-1^ | $\hat{\boldsymbol{q}}$**_NO3-_**  gN gCOD^-1^d^-1^ | **K_NO3-_**  mgN L^-1^ | **Source** |
| --- | --- | --- | --- | --- | --- |
| *Paracoccus pantotrophus* | 2.7 | 0.93 | 2.9 | - | This study |
| Mixed culture | 0.47^*^ | 0.5^*^ | 0.94 | 0.025 | Pan, Ni and Yuan, (2013) |
| Mixed culture | 1.27^*^ | 0.67^*^ | 0.75 | 0.251 | Ni et al., (2011) |
| Mixed culture | 0.552^*^ | 0.6^*^ | 0.92 | 0.2 | Hiatt and Grady, (2008) |
| Mixed culture | - | 0.67^*^ | - | 0.5 | Henze, (2000) |
| Mixed culture | - | 0.65^*^ | - | 0.5 | Koch et al. (2000) |
| Mixed culture | - | 0.67^*^ | - | 0.5 | Henze et al., (1999) |
| Mixed culture | - | - | 0.22 | 1.12 | Wicht (1996) |
| Mixed culture | - | - | 1.14, 1.76 | 0.247, 0.713 | Wild, von Schulthess and Gujer (1995) |
| Mixed culture | - | - | 1.27, 1.14 | 0.251, 0.247 | Wild, von Schulthess and Gujer (1994) |
| Mixed culture | - | 0.67 | - | - | Gujer W. and Henze M. (1991) |
| Mixed culture | - | - | - | 0.2 | George Tchobanoglous and Franklin L Burton and Metcalf & Eddy, (1991) |
| **calculated parameters* |  |  |  |  |  |

**Table S4.** Kinetic parameters for NO_2_^-^ to N_2_O reduction by mixed and pure cultures of denitrifying bacteria

**calculated parameters*

| **Type of**  **Bacteria** | $\hat{\boldsymbol{\mu}}$***_NO2-_***  d^-1^ | **Y_NO2-_**  g COD gN^-1^ | $\hat{\boldsymbol{q}}$***_NO2-_***  gN gCOD^-1^ d^-1^ | **K_NO2-_**  mgN L^-1^ | **Source** |
| --- | --- | --- | --- | --- | --- |
| *Paracoccus pantotrophus* | 0.93 | 0.65 | 1.4 | - | This study |
| Mixed culture | 0.625* | 0.5* | 1.25 | - | Pan, Ni and Yuan (2013) |
| Mixed culture | 1.34* | 0.67* | 2* | - | Ni et al. (2011) |
| Mixed culture | - | 0.6* | - | 0.2* | Hiatt and Grady (2008) |
| Mixed culture | - | 0.5 | - | 0.5 | Koch et al. (2000) |
| Mixed culture | - | - | - | 0.058 | Henze et al. (1999) |
| Mixed culture | - | - | 0.18 | 0.23 | Wicht (1996) |
| Mixed culture | - | - | 1.16, 1.70 | 2.37, 3.50 | Wild, von Schulthess and Gujer (1995) |
| Mixed culture | - | - | 1.34, 1.16 | 0.81, 2.37 | von Schulthess, Wild and Gujer (1994) |

**Table S5.** Kinetic parameters for N_2_O to N_2_ reduction by mixed and pure cultures of denitrifying bacteria

| **Type of Bacteria** | $\hat{\boldsymbol{\mu}}$***_N2O_***  d^-1^ | **Y_N2O_**  gCOD gN^-1^ | $\hat{\boldsymbol{q}}$***_N2O_***  gN gCOD^-1^ d^-1^ | **K_N2O_**  mgN L^-1^ | **Source** |
| --- | --- | --- | --- | --- | --- |
| *Paracoccus pantotrophus* | 1.7 | 0.32 | 5.3 | - | This study |
| Mixed culture | 2.36* | 0.5* | 4.73 | 0.35 | Pan, Ni and Yuan (2013) |
| Mixed culture | 3.22* | 0.67* | 2.41 |  | Ni et al. (2011) |
| Mixed culture | - | 0.6* | - | 0.05* | Hiatt and Grady (2008) |
| *Paracoccus denitrificans* | - | 0.21-0.29 | - | - | Strohm et al. (2007) |
| *Pseudomonas stutzeri* | - | 0.50 | - | - | Strohm et al. (2007) |
| Mixed culture | - | - | - | 0.0028,  0.0112 | (Holtan-Hartwig, Dorsch and Bakken, 2000) |
| Mixed culture | - | - | 0.88 | 0.035 | Wicht (1996) |
| Mixed culture | - | - | 3.02, 11.1 | 0.0095, 0.0262 | Wild, von Schulthess and Gujer (1995) |
| Mixed culture | - | - | 3.21, 3.02 | 0.0052, 0.0095 | von Schulthess, Wild and Gujer (1994) |
| *Alcaligenes faecalis* | - | 1.14 | - | - | Okereke (1993) |
| *Pseudomonas stutzeri* | - | 0.83 | - | - | Okereke (1993) |
| *Paracoccus denitrificans* | - | 0.62 | - | - | Okereke (1993) |
| *Pseudomonas perfectomarinus* | - | 0.45 | - | - | Okereke (1993) |
| *Pseudomonas denitrificans* | 1.37- 2.57 | 0.8 | - | - | Koike and Hattori (1975) |
| **calculated parameters* |  |  |  |  |  |

**REFERENCES**

George Tchobanoglous and Franklin L. Burton and Metcalf & Eddy (1991) Wastewater engineering: treatment, disposal and reuse. McGraw-Hill, New York, .

Gujer W. and Henze M. 1991. Activated sludge modeling and simulation. Water Science and Technology 23 (4-6), 1011-1023.

Hellinga, C., van Loosdrecht, M., Heijnen, J. 1999. Model based design of a novel process for nitrogen removal from concentrated flows. Mathematical and Computer Modelling of Dynamical Systems 5 (4), 351-371.

Henze, M., Gujer, W., Mino, T., Matsuo, T., Wentzel, M. C., Marais, G. v. R., van Loosdrecht, M. C. M. 1999. Activated sludge model No.2D, ASM2D. Water Science and Technology 39 (1), 165-182.

Henze, M. (2000) Activated sludge models ASM1, ASM2, ASM2d and ASM3. IWA Scientific and Technical Report No. 9, London

Hiatt, W. C. and Grady, C. P. L., Jr. 2008. An updated process model for carbon oxidation, nitrification, and denitrification. Water Environment Research 80 (11), 2145-2156.

Holtan-Hartwig, L., Dorsch, P., Bakken, L. 2000. Comparison of denitrifying communities in organic soils: kinetics of NO3- and N2O reduction. Soil Biology & Biochemistry 32 (6), 833-843.

Koch, G., Kühni, M., Gujer, W., Siegrist, H. 2000. Calibration and validation of activated sludge model no. 3 for Swiss municipal wastewater. Water Research 34 (14), 3580-3590.

Koike, I. and Hattori, A. 1975. Energy yield of denitrification - Estimate from growth yield in continuous cultures of Pseudomonas denitrificans under nitrate-limited, nitrite-limited and nitrous oxide-limited conditions. Journal of General Microbiology 88 (May), 11-19.

Ni, B., Ruscalleda, M., Pellicer-Nacher, C., Smets, B. F. 2011. Modeling nitrous oxide production during biological nitrogen removal via nitrification and denitrification: extensions to the general ASM models. Environmental Science & Technology 45 (18), 7768-7776.

Okereke, G. U. 1993. Growth yield of denitrifiers using nitrous oxide as a terminal electron acceptor. World Journal of Microbiology & Biotechnology 9 (1), 59-62.

Pan, Y., Ni, B., Yuan, Z. 2013. Modeling electron competition among nitrogen oxides reduction and N2O accumulation in denitrification. Environmental Science & Technology 47 (19), 11083-11091.

Rittmann, B. E. and McCarty, P. L. (2001) Environmental Biotechnology: Principles and Applications. McGraw-Hill Book Co, New York, .

Strohm, T. O., Griffin, B., Zumft, W. G., Schink, B. 2007. Growth yields in bacterial denitrification and nitrate ammonification. Applied and Environmental Microbiology 73 (5), 1420-1424.

von Schulthess, R., Wild, D., Gujer, W. 1994. Nitric and nitrous oxides from denitrifying activated sludge at low-oxygen concentration. Water Science and Technology 30 (6), 123-132.

von Schulthess, R., Kuhni, M., Gujer, R. 1995. Release of nitric and nitrous oxides from denitrifying activated-sludge. Water Research 29 (1), 215-226.

von Schulthess, R. and Gujer, W. 1996. Release of nitrous oxide (N2O) from denitrifying activated sludge: verification and application of a mathematical model. Water Research 30 (3), 521-530.

Wild, D., von Schulthess, R., Gujer, W. 1994. Synthesis of denitrification enzymes in activated-sludge - modeling with structured biomass. Water Science and Technology 30 (6), 113-122.

Wild, D., von Schulthess, R., Gujer, W. 1995. Structured modeling of denitrification intermediates. Water Science and Technology 31 (2), 45-54.

Wicht, H. 1996. A model for predicting nitrous oxide production during denitrification in activated sludge. Water Science and Technology 34 (5-6), 99-106.
